# Nomenclature for the KIR of non-human species

**DOI:** 10.1007/s00251-018-1064-4

**Published:** 2018-06-04

**Authors:** James Robinson, Lisbeth A. Guethlein, Giuseppe Maccari, Jeroen Blokhuis, Benjamin N. Bimber, Natasja G. de Groot, Nicholas D. Sanderson, Laurent Abi-Rached, Lutz Walter, Ronald E. Bontrop, John A. Hammond, Steven G. E. Marsh, Peter Parham

**Affiliations:** 1 Anthony Nolan Research Institute, London, UK; 2 UCL Cancer Institute, University College London, London, UK; 3 Department of Structural Biology and Department of Microbiology & Immunology, School of Medicine, Stanford University, Stanford, CA USA; 4 The Pirbright Institute, Woking, UK; 5 Biomedical Primate Research Centre, Rijswijk, Netherlands; 6 Parker Institute for Cancer Immunotherapy, San Francisco, CA USA; 7 Oregon National Primate Research Centre, Oregon Health and Science University, OR Beaverton, USA; 8 Nuffield Department of Clinical Medicine, University of Oxford, Oxford, UK; 9 Aix-Marseille II University, Marseille, France; 10 German Primate Centre, Gottingen, Germany

**Keywords:** KIR, Nomenclature, Variant, Allele, Gene, Database, Sequence

## Abstract

The increasing number of Killer Immunoglobulin-like Receptor (KIR) sequences available for non-human primate species and cattle has prompted development of a centralized database, guidelines for a standardized nomenclature, and minimum requirements for database submission. The guidelines and nomenclature are based on those used for human KIR and incorporate modifications made for inclusion of non-human species in the companion IPD-NHKIR database. Included in this first release are the rhesus macaque (*Macaca mulatta*), chimpanzee (*Pan troglodytes*), orangutan (*Pongo abelii* and *Pongo pygmaeus*), and cattle (*Bos taurus*).

## Introduction

The *KIR* locus has been studied in a number of non-human species primates and is characterized by high levels of allelic polymorphism, haplotypic polymorphism in the number of genes, and extensive duplication and recombination (Hammond et al. 2016; Parham 2004). These factors have made it difficult to assign orthologues and have led to a number of different nomenclature systems being used to name genes and alleles. This report describes a common framework and guidelines for KIR nomenclature in non-human species. These have been developed by taking advantage of lessons learned in the development of a nomenclature system for the human KIR (Marsh et al. 2003).

### General naming guidelines

To provide consistency with the IPD-MHC Database (Maccari et al. 2017), the non-human KIR nomenclature adopts the same four-character prefix used for species designation in the naming of *MHC* alleles (de Groot et al. 2012; Ellis et al. 2006; Klein et al. 1990). Also, genes and alleles will be named based on the conventions that have been adopted for the human KIR system (Marsh et al. 2003) that are based on the structures of the molecules they encode. The first digit following the KIR acronym corresponds to the number of Ig-like domains in the polypeptide and the “D” denotes “Domain.” The D is followed by either an “L” indicating a “Long” cytoplasmic tail, an “S” indicating a “Short” cytoplasmic tail or a “P” for pseudogenes. In addition, the inclusion of a “W” indicates “Workshop” following the “L,” “S,” or “P” to indicate any sequence that by phylogenetic analysis is sufficiently divergent to be considered a “new” gene, but lack either genomic sequencing or family studies to demonstrate that it does define a new gene and not a divergent lineage a known gene. Tables 1, 2, and 3 list the current gene designations and their previous names. Symbols for genes are italicized (e.g., *Mamu*-*KIR3DL01*), whereas symbols for proteins are not italicized (e.g., Mamu-KIR3DL01). Alleles follow the same conventions as gene names.

**Table 1:** Gene designations and their previous names

Species	KIR gene designation(s)	Previous KIR gene designation(s)
Rhesus macaque (Mamu)	*Mamu*-*KIR1D*	KIR1D, Mamu-KIR1D
	*Mamu*-*KIR2DL04*	2DL501NK, 2DL503NK, KIR2DL4, KIR2DL4.1, MmKIR2DL4
	*Mamu*-*KIR3DL01*	2DL426NK, 3DL34, KIR3DL, KIR3DL-like_1, KIR3DL1, KIR3DL1-like1, KIR3DL12, KIR3DL13, KIR3DL14, KIR3DL15, KIR3DL19, KIR3DL1_variant_2, KIR3DL2, KIR3DL2-old, KIR3DL3, KIR3DL4, KIR3DL5
	*Mamu*-*KIR3DL02*	KIR3DL-like_3, KIR3DL2, KIR3DL21, KIR3DL21-like1
	*Mamu*-*KIR3DL04*	KIR3DL11
	*Mamu*-*KIR3DL05*	3DL7b-3DL40, KIR3DL, KIR3DL-3, KIR3DL16, KIR3DL7, KIR3DL7-like2, KIR3DL07
	*Mamu*-*KIR3DL06*	KIR3DL6
	*Mamu*-*KIR3DL07*	2DL420, KIR3DL, KIR3DL18, KIR3DL7, KIR3DL7-like1, KIR3DL7-like3, KIR3DL03
	*Mamu*-*KIR3DL08*	KIR3DL, KIR3DL-like_2, KIR3DL17, KIR3DL8, KIRDL8, Mamu-KIR3DL04, Mamu-KIR3DL4
	*Mamu*-*KIR3DL10*	3DL10-2DL501, 3DL3NK, KIR3DL, KIR3DL10, KIR3DL9, Mamu-KIR3DL05
	*Mamu*-*KIR3DL11*	KIR3DL, KIR3DL-1, KIR3DL-6, KIR3DL-7, KIR3DL11
	*Mamu*-*KIR3DL20*	KIR3DL20, KIR3DL20_variant_2, KIR3DL06, KIR2DL5
	*Mamu*-*KIR3DLW03*	KIR3DL-4, KIR3DL-5, KIR3DL-like1-BNB, KIR3DL21
	*Mamu*-*KIR3DLX1*	KIR3DL0
	*Mamu*-*KIR3DS01*	KIR3DH-7, KIR3DH1, KIR3DH5, Mamu-KIR3DS01-JHB-HEFGH,
	*Mamu*-*KIR3DS02*	3DH2, 3DH42, KIR3DH-like_5, KIR3DH-like_6, KIR3DH10, KIR3DH12, KIR3DH13, KIR3DH14, KIR3DH15, KIR3DH16, KIR3DH2
	*Mamu*-*KIR3DS03*	KIR3DH3, KIR3DH8, KIR3DH9
	*Mamu*-*KIR3DS04*	KIR3DH-1, KIR3DH4, KIR3DH6
	*Mamu*-*KIR3DS05*	KIR3DH1, KIR3DM-1, KIR3DM1, KIR3DM6, KIR_Partial_Sequence_1
	*Mamu*-*KIR3DS06*	KIR3DH-4, KIR3DH-like8, KIR3DH-like_7, KIR3DH18,
	*Mamu*-*KIR3DSW07*	KIR3DH-5, KIR3DH7, Mamu-KIR3DS07-JHB-HO
	*Mamu*-*KIR3DSW08*	KIR3DH-2, KIR3DH-3, KIR3DH-4, KIR3DH-5, KIR3DH-like_1, KIR3DH-like_2, KIR3DH-like_3, KIR3DH-like_4, KIR3DH21, KIR3DSW08
	*Mamu*-*KIR3DSW09*	KIR3DH-8, KIR3DH20, KIR3DH5, KIR3DH5-like1, mmKIR3DH-1

**Table 2:** Gene designations and their previous names

Species	KIR gene designation(s)	Previous KIR gene designation(s)
Chimpanzee (Patr)	*Patr*-*KIR2DL4*	
	*Patr*-*KIR2DL5*	
	*Patr*-*KIR2DL6*	Pt-NewII
	*Patr*-*KIR2DL7*	
	*Patr*-*KIR2DL8*	Pt-NewIII
	*Patr*-*KIR2DL9*	
	*Patr*-*KIR3DL1*	Pt-KIR3DL1/2, Pt-KIR3DL3, Pt-KIR3DL1, Pt-KIR3DL2
	*Patr*-*KIR3DL3*	Patr-KIRC1, Pt-NewI
	*Patr*-*KIR3DL4*	
	*Patr*-*KIR3DL5*	
	*Patr*-*KIR3DS6*	Pt-KIR3DL6

**Table 3:** Gene designations and their previous names

Species	KIR gene designation(s)	Previous KIR gene designation(s)
Orangutan (Poab)	*Poab*-*KIR2DL10*	Popy-KIR2DL10, 2DLA
	*Poab*-*KIR2DL11*	Popy-KIR2DL11, 2DLB
	*Poab*-*KIR2DL12*	Popy-KIR2DL11, 2DLC
	*Poab*-*KIR2DL5*	Popy-KIR2DL5. 2DL5
	*Poab*-*KIR2DS10*	2DSD/2DSA
	*Poab*-*KIR2DS13*	Popy-KIR2DS13, 2DSC1/2DSB
	*Poab*-*KIR2DS14*	Popy-KIR2DS14, 2DSB/2DSD2, 2DSA/2DSD1
	*Poab*-*KIR3DL1*	Popy-KIR3DL1, 3DLH, 3DLC, 3DLD2, 3DLD1, 3DLA, 3DLI, 3DLB
	*Poab*-*KIR3DL3*	Popy-KIR3DL3, 3DL3
	*Poab*-*KIR3DS1*	Popy-KIR3DS1, 3DS1
	*Poab*-*KIRDP*	Popy-KIRDP, DP
Orangutan (Popy)	*Popy*-*KIR2DL11*	Popy-KIR2DLB
	*Popy*-*KIR2DL12*	Popy-KIR2DLC
	*Popy*-*KIR2DL5*	
	*Popy*-*KIR2DS10*	Popy-KIR2DSD/2DSA
	*Popy*-*KIR2DS13*	Popy-KIR2DSC2/2DSB
	*Popy*-*KIR2DS14*	Popy-KIR2DSB/2DSD2, 2DSA/2DSD1
	*Popy*-*KIR2DS15*	
	*Popy*-*KIR3DL1*	Popy-KIR3DL1, 3DLF, 3DLE2, 3DLE1
	*Popy*-*KIR3DL3*	Popy-KIR3DL3, 3DL3
	*Popy*-*KIR3DS1*	Popy-KIR3DS1, 3DS1
	*Popy*-*KIRDP*	Popy-KIRDP, DP

Reflecting species-specific differences, there have been further additions/modifications to the general nomenclature for rhesus macaque and cattle. As with the human KIR nomenclature, alleles in each series have been named in order of their deposition into a generalist sequence databank, GenBank/EMBL-ENA/DDBJ (Benson et al. 2017; Chojnacki et al. 2017; Mashima et al. 2017). Where the identity is known of the animal providing the sequenced DNA, that information is included in the database, as well as information regarding the origin of the animal. Tables 4, 5, 6, and 7 provide a complete list of genes and alleles currently in the nomenclature, as well as the original name(s), accession number, and reference to the original report of the sequence.

**Table 4:** Allele designations and their previous names

Gene	Allele designation	Previous designations	Accession number	Reference
*Mamu*-*KIR1D*	*Mamu*-KIR1D*001	KIR1D	AF334634	(Hershberger et al. 2001)
*Mamu*-*KIR1D*	*Mamu*-KIR1D*002	KIR1D,Mamu-KIR1D*00202-JHB-HA	AY728181, GU112257, GU112266, GU112332	(Sambrook et al. 2005) (Blokhuis et al. 2010)
*Mamu*-*KIR2DL04*	*Mamu*-KIR2DL04*001:01	KIR2DL4, KIR2DL4.1, MmKIR2DL4*0010101-JHB	EU702486, AF361088, AF334644, FJ824091, GU112331, GU112318, GU112263, GU112303, GU112287	(Blokhuis et al. 2009a; Blokhuis et al. 2009b; Blokhuis et al. 2010; Grendell et al. 2001; Hershberger et al. 2001)
*Mamu*-*KIR2DL04*	*Mamu*-KIR2DL04*001:02	2DL501NK	GU299490	(Colantonio et al. 2011)
*Mamu*-*KIR2DL04*	*Mamu*-KIR2DL04*002	MmKIR2DL4*0020101-JHB	FJ824092, GU112279	(Blokhuis et al. 2009b; Blokhuis et al. 2010)
*Mamu*-*KIR2DL04*	*Mamu*-KIR2DL04*003	KIR2DL4, MmKIR2DL4*0040101-JHB	AY505486, FJ824093, GU112322, GU112284	(Andersen et al. 2004; Blokhuis et al. 2009b; Blokhuis et al. 2010)
*Mamu*-*KIR2DL04*	*Mamu*-KIR2DL04*004	KIR2DL4	AY728182	(Sambrook et al. 2005)
*Mamu*-*KIR2DL04*	*Mamu*-KIR2DL04*005	MmKIR2DL4*0050101-JHB	FJ824094	(Blokhuis et al. 2009b)
*Mamu*-*KIR2DL04*	*Mamu*-KIR2DL04*006:01	MmKIR2DL4*0060101-JHB	FJ824095	(Blokhuis et al. 2009b)
*Mamu*-*KIR2DL04*	*Mamu*-KIR2DL04*006:02	2DL503NK	GU014298	(Colantonio et al. 2011)
*Mamu*-*KIR2DL04*	*Mamu*-KIR2DL04*007	MmKIR2DL4*0070101-JHB	FJ824096	(Blokhuis et al. 2009b)
*Mamu*-*KIR2DL04*	*Mamu*-KIR2DL04*008:01	MmKIR2DL4*0080101-JHB	FJ824097	(Blokhuis et al. 2009b)
*Mamu*-*KIR2DL04*	*Mamu*-KIR2DL04*008:02	MmKIR2DL4*0080201-JHB	FJ824098, GU112326	(Blokhuis et al. 2009b; Blokhuis et al. 2010)
*Mamu*-*KIR2DL04*	*Mamu*-KIR2DL04*010	MmKIR2DL4*0100101-JHB	FJ824100	(Blokhuis et al. 2009b)
*Mamu*-*KIR2DL04*	*Mamu*-KIR2DL04*011	MmKIR2DL4*0110101-JHB	FJ824101	(Blokhuis et al. 2009b)
*Mamu*-*KIR2DL04*	*Mamu*-KIR2DL04*012	MmKIR2DL4*0120101-JHB	FJ824102	(Blokhuis et al. 2009b)
*Mamu*-*KIR2DL04*	*Mamu*-KIR2DL04*013	MmKIR2DL4*0130101-JHB	FJ824103	(Blokhuis et al. 2009b)
*Mamu*-*KIR2DL04*	*Mamu*-KIR2DL04*014:01	MmKIR2DL4*0140101-JHB	FJ824104, GU112316	(Blokhuis et al. 2009b; Blokhuis et al. 2010)
*Mamu*-*KIR2DL04*	*Mamu*-KIR2DL04*014:02	MmKIR2DL4*0140201-JHB	FJ824105	(Blokhuis et al. 2009b)
*Mamu*-*KIR2DL04*	*Mamu*-KIR2DL04*015:01	MmKIR2DL4*0150101-JHB	FJ824106, GU112313	(Blokhuis et al. 2009b; Blokhuis et al. 2010)
*Mamu*-*KIR2DL04*	*Mamu*-KIR2DL04*015:02	MmKIR2DL4*0150201-JHB	FJ824107, GU112280	(Blokhuis et al. 2009b; Blokhuis et al. 2010)
*Mamu*-*KIR2DL04*	*Mamu*-KIR2DL04*016	MmKIR2DL4*0160101-JHB	FJ824108	(Blokhuis et al. 2009b)
*Mamu*-*KIR2DL04*	*Mamu*-KIR2DL04*017	MmKIR2DL4*0170101-JHB	FJ824109	(Blokhuis et al. 2009b)
*Mamu*-*KIR2DL04*	*Mamu*-KIR2DL04*018	MmKIR2DL4*0180101-JHB	FJ824110	(Blokhuis et al. 2009b)
*Mamu*-*KIR2DL04*	*Mamu*-KIR2DL04*019	MmKIR2DL4*0190101-JHB	FJ824111	(Blokhuis et al. 2009b)
*Mamu*-*KIR2DL04*	*Mamu*-KIR2DL04*020	MmKIR2DL4*0200101-JHB	FJ824112, GU112274	(Blokhuis et al. 2009b; Blokhuis et al. 2010)
*Mamu*-*KIR3DL01*	*Mamu*-KIR3DL01*001	KIR3DL1, 3DL34	AF334616, GU299488	(Colantonio et al. 2011; Hershberger et al. 2001)
*Mamu*-*KIR3DL01*	*Mamu*-KIR3DL01*002	KIR3DL2-old, 2DL426NK	AF334617, GU299488	(Hershberger et al. 2001), (Colantonio et al. 2011)
*Mamu*-*KIR3DL01*	*Mamu*-KIR3DL01*003	KIR3DL3	AF361083, GU112305	(Blokhuis et al. 2010; Grendell et al. 2001)
*Mamu*-*KIR3DL01*	*Mamu*-KIR3DL01*004	KIR3DL4	AF334619	(Hershberger et al. 2001)
*Mamu*-*KIR3DL01*	*Mamu*-KIR3DL01*005	KIR3DL5	AF334620	(Hershberger et al. 2001)
*Mamu*-*KIR3DL01*	*Mamu*-KIR3DL01*006	KIR3DL12	AF361082	(Grendell et al. 2001)
*Mamu*-*KIR3DL01*	*Mamu*-KIR3DL01*007N	KIR3DL13	AF408151	(Grendell et al. 2001)
*Mamu*-*KIR3DL01*	*Mamu*-KIR3DL01*008N	KIR3DL14	AF408152	(Grendell et al. 2001)
*Mamu*-*KIR3DL01*	*Mamu*-KIR3DL01*009N	KIR3DL15	AF408153	(Grendell et al. 2001)
*Mamu*-*KIR3DL01*	*Mamu*-KIR3DL01*010	KIR3DL19	AF408150	(Grendell et al. 2001)
*Mamu*-*KIR3DL01*	*Mamu*-KIR3DL01*011	KIR3DL1_variant_2	AY728187	(Sambrook et al. 2005)
*Mamu*-*KIR3DL01*	*Mamu*-KIR3DL01*012	KIR3DL1*002-BNB, KIR3DL-like_1	EU419033, AY505476, GU112286	(Andersen et al. 2004; Blokhuis et al. 2010; Moreland et al. 2011)
*Mamu*-*KIR3DL01*	*Mamu*-KIR3DL01*013	KIR3DL1*003-BNB	EU419034	(Moreland et al. 2011)
*Mamu*-*KIR3DL01*	*Mamu*-KIR3DL01*014	KIR3DL1*005-BNB	EU419035	(Moreland et al. 2011)
*Mamu*-*KIR3DL01*	*Mamu*-KIR3DL01*015	KIR3DL1*006-BNB	EU419036	(Moreland et al. 2011)
*Mamu*-*KIR3DL01*	*Mamu*-KIR3DL01*016	KIR3DL1*007-BNB	EU419037, GU112258	(Blokhuis et al. 2010; Moreland et al. 2011)
*Mamu*-*KIR3DL01*	*Mamu*-KIR3DL01*017	KIR3DL12*001-BNB	EU419044	(Moreland et al. 2011)
*Mamu*-*KIR3DL01*	*Mamu*-KIR3DL01*018	KIR3DL2*001-BNB	EU419046	(Moreland et al. 2011)
*Mamu*-*KIR3DL01*	*Mamu*-KIR3DL01*019:01	KIR3DL1*001-BNB	EU419032, GU112300	(Blokhuis et al. 2010; Moreland et al. 2011)
*Mamu*-*KIR3DL01*	*Mamu*-KIR3DL01*019:02	None	GU112283	(Blokhuis et al. 2010)
*Mamu*-*KIR3DL01*	*Mamu*-KIR3DL01*020	KIR3DL1-like1	EU688987	(Moreland et al. 2011)
*Mamu*-*KIR3DL01*	*Mamu*-KIR3DL01*021	KIR3DL	FJ562108	(Bostik et al. 2009)
*Mamu*-*KIR3DL01*	*Mamu*-KIR3DL01*022	None	GU112267	(Blokhuis et al. 2010)
*Mamu*-*KIR3DL01*	*Mamu*-KIR3DL01*023	None	GU112292	(Blokhuis et al. 2010)
*Mamu*-*KIR3DL01*	*Mamu*-KIR3DL01*024	None	GU112321	(Blokhuis et al. 2010)
*Mamu*-*KIR3DL01*	*Mamu*-KIR3DL01*025	None	GU112324	(Blokhuis et al. 2010)
*Mamu*-*KIR3DL01*	*Mamu*-KIR3DL01*026	KIR3DL allele 2	FJ562109	(Bostik et al. 2009)
*Mamu*-*KIR3DL01*	*Mamu*-KIR3DL01*027	KIR3DL allele 3	FJ562110	(Bostik et al. 2009)
*Mamu*-*KIR3DL02*	*Mamu*-KIR3DL02*001	KIR3DL2	AY728188	(Sambrook et al. 2005)
*Mamu*-*KIR3DL02*	*Mamu*-KIR3DL02*002	KIR3DL-like_3	AY505478	(Andersen et al. 2004)
*Mamu*-*KIR3DL02*	*Mamu*-KIR3DL02*003	KIR3DL21*001-BNB	EU419050	(Moreland et al. 2011)
*Mamu*-*KIR3DL02*	*Mamu*-KIR3DL02*004:01	KIR3DL21*003-BNB	EU419052	(Moreland et al. 2011)
*Mamu*-*KIR3DL02*	*Mamu*-KIR3DL02*004:02	KIR3DL21*005-BNB	EU419053	(Moreland et al. 2011)
*Mamu*-*KIR3DL02*	*Mamu*-KIR3DL02*005	KIR3DL21*006-BNB	EU419054	(Moreland et al. 2011)
*Mamu*-*KIR3DL02*	*Mamu*-KIR3DL02*006	KIR3DL21-like1	EU688989	(Moreland et al. 2011)
*Mamu*-*KIR3DL02*	*Mamu*-KIR3DL02*007	None	GU112277	(Blokhuis et al. 2010)
*Mamu*-*KIR3DL02*	*Mamu*-KIR3DL02*008	None	GU112281	(Blokhuis et al. 2010)
*Mamu*-*KIR3DLW03*	*Mamu*-KIR3DLW03*001	KIR3DL21*002-BNB	EU419051	(Moreland et al. 2011)
*Mamu*-*KIR3DLW03*	*Mamu*-KIR3DLW03*002	KIR3DL21*007-BNB	EU419055	(Moreland et al. 2011)
*Mamu*-*KIR3DLW03*	*Mamu*-KIR3DLW03*003	KIR3DL-like1-BNB	EU419031	(Moreland et al. 2011)
*Mamu*-*KIR3DLW03*	*Mamu*-KIR3DLW03*004	KIR3DL-4	FN424253	(Kruse et al. 2010)
*Mamu*-*KIR3DLW03*	*Mamu*-KIR3DLW03*005	KIR3DL-5	FN424256	(Kruse et al. 2010)
*Mamu*-*KIR3DL04*	*Mamu*-KIR3DL04*001:01	KIR3DL11*002-BNB	EU419040	(Moreland et al. 2011)
*Mamu*-*KIR3DL04*	*Mamu*-KIR3DL04*001:02	None	GU112311	(Blokhuis et al. 2010)
*Mamu*-*KIR3DL04*	*Mamu*-KIR3DL04*001:03	None	GU112319	(Blokhuis et al. 2010)
*Mamu*-*KIR3DL04*	*Mamu*-KIR3DL04*002	KIR3DL11*003-BNB	EU419042	(Moreland et al. 2011)
*Mamu*-*KIR3DL05*	*Mamu*-KIR3DL05*001	KIR3DL16*001-BNB	EU419045	(Moreland et al. 2011)
*Mamu*-*KIR3DL05*	*Mamu*-KIR3DL05*002	KIR3DL7*004-BNB	EU419061	(Moreland et al. 2011)
*Mamu*-*KIR3DL05*	*Mamu*-KIR3DL05*003	KIR3DL7*005-BNB	EU419062	(Moreland et al. 2011)
*Mamu*-*KIR3DL05*	*Mamu*-KIR3DL05*004	KIR3DL7*009-BNB	EU419066	(Moreland et al. 2011)
*Mamu*-*KIR3DL05*	*Mamu*-KIR3DL05*005	KIR3DL7*013-BNB	EU419069	(Moreland et al. 2011)
*Mamu*-*KIR3DL05*	*Mamu*-KIR3DL05*006:01	KIR3DL7-like2	EU688991	(Moreland et al. 2011)
*Mamu*-*KIR3DL05*	*Mamu*-KIR3DL05*006:02	None	GU112293	(Blokhuis et al. 2010)
*Mamu*-*KIR3DL05*	*Mamu*-KIR3DL05*007	KIR3DL-3	FN424252	(Kruse et al. 2010)
*Mamu*-*KIR3DL05*	*Mamu*-KIR3DL05*008	3DL7b-3DL40	GU112291, GU014295	(Blokhuis et al. 2010) (Colantonio et al. 2011)
*Mamu*-*KIR3DL05*	*Mamu*-KIR3DL05*009	None	GU112310	(Blokhuis et al. 2010)
*Mamu*-*KIR3DL05*	*Mamu*-KIR3DL05*010	KIR3DL allele 13	FJ562120	(Bostik et al. 2009)
*Mamu*-*KIR3DL05*	*Mamu*-KIR3DL05*011	KIR3DL allele 14	FJ562121	(Bostik et al. 2009)
*Mamu*-*KIR3DL06*	*Mamu*-KIR3DL06*001	KIR3DL6	AF334621	(Hershberger et al. 2001)
*Mamu*-*KIR3DL06*	*Mamu*-KIR3DL06*002	KIR3DL6*001-BNB	EU419056	(Moreland et al. 2011)
*Mamu*-*KIR3DL07*	*Mamu*-KIR3DL07*001	KIR3DL7	AF334622	(Hershberger et al. 2001)
*Mamu*-*KIR3DL07*	*Mamu*-KIR3DL07*002	KIR3DL18	AF361086	(Grendell et al. 2001)
*Mamu*-*KIR3DL07*	*Mamu*-KIR3DL07*003	KIR3DL7*001-BNB	EU419057	(Moreland et al. 2011)
*Mamu*-*KIR3DL07*	*Mamu*-KIR3DL07*004	KIR3DL7*003-BNB	EU419060	(Moreland et al. 2011)
*Mamu*-*KIR3DL07*	*Mamu*-KIR3DL07*005	KIR3DL7*006-BNB	EU419063	(Moreland et al. 2011)
*Mamu*-*KIR3DL07*	*Mamu*-KIR3DL07*006	KIR3DL7*007-BNB	EU419064	(Moreland et al. 2011)
*Mamu*-*KIR3DL07*	*Mamu*-KIR3DL07*007	KIR3DL7*008-BNB	EU419065	(Moreland et al. 2011)
*Mamu*-*KIR3DL07*	*Mamu*-KIR3DL07*008	KIR3DL7*012-BNB	EU419068	(Moreland et al. 2011)
*Mamu*-*KIR3DL07*	*Mamu*-KIR3DL07*009:01	KIR3DL7-like1, 2DL420	EU688990, GU299489	(Colantonio et al. 2011; Moreland et al. 2011)
*Mamu*-*KIR3DL07*	*Mamu*-KIR3DL07*009:02	None	GU112282	(Blokhuis et al. 2010)
*Mamu*-*KIR3DL07*	*Mamu*-KIR3DL07*010	KIR3DL7-like3	EU688992	(Moreland et al. 2011)
*Mamu*-*KIR3DL07*	*Mamu*-KIR3DL07*011	KIR3DL allele 10	FJ562117	(Bostik et al. 2009)
*Mamu*-*KIR3DL07*	*Mamu*-KIR3DL07*012	KIR3DL allele 11	FJ562118	(Bostik et al. 2009)
*Mamu*-*KIR3DL08*	*Mamu*-KIR3DL08*001:01	KIR3DL8	AY728189	(Sambrook et al. 2005)
*Mamu*-*KIR3DL08*	*Mamu*-KIR3DL08*001:02	KIR3DL8*002-BNB	EU419071	(Moreland et al. 2011)
*Mamu*-*KIR3DL08*	*Mamu*-KIR3DL08*002	KIR3DL17	AF361084, GU112306	(Blokhuis et al. 2010; Grendell et al. 2001)
*Mamu*-*KIR3DL08*	*Mamu*-KIR3DL08*003	KIR3DL17	AF361085	(Grendell et al. 2001)
*Mamu*-*KIR3DL08*	*Mamu*-KIR3DL08*004	KIR3DL-like_2	AY505477	(Andersen et al. 2004)
*Mamu*-*KIR3DL08*	*Mamu*-KIR3DL08*005	KIRDL8	AY728189	(Sambrook et al. 2005)
*Mamu*-*KIR3DL08*	*Mamu*-KIR3DL08*006	KIR3DL8*001-BNB	EU419070	(Moreland et al. 2011)
*Mamu*-*KIR3DL08*	*Mamu*-KIR3DL08*007	None	GU112268	(Blokhuis et al. 2010)
*Mamu*-*KIR3DL08*	*Mamu*-KIR3DL08*008	None	GU112285	(Blokhuis et al. 2010)
*Mamu*-*KIR3DL08*	*Mamu*-KIR3DL08*009	None	GU112290	(Blokhuis et al. 2010)
*Mamu*-*KIR3DL08*	*Mamu*-KIR3DL08*010	None	GU112330	(Blokhuis et al. 2010)
*Mamu*-*KIR3DL08*	*Mamu*-KIR3DL08*011	KIR3DL allele 8	FJ562115	(Bostik et al. 2009)
*Mamu*-*KIR3DL10*	*Mamu*-KIR3DL10*001	KIR3DL10	AY728183	(Sambrook et al. 2005)
*Mamu*-*KIR3DL10*	*Mamu*-KIR3DL10*002:01	KIR3DL9, KIR3DL allele 5	AF334624, GU112259, FJ562112	(Hershberger et al. 2001)(Blokhuis et al. 2010; Bostik et al. 2009)
*Mamu*-*KIR3DL10*	*Mamu*-KIR3DL10*002:02	3DL3NK	GU299486	(Colantonio et al. 2011)
*Mamu*-*KIR3DL10*	*Mamu*-KIR3DL10*003	KIR3DL10*001-BNB	EU419038	(Moreland et al. 2011)
*Mamu*-*KIR3DL10*	*Mamu*-KIR3DL10*004	KIR3DL10*002-BNB	EU419039	(Moreland et al. 2011)
*Mamu*-*KIR3DL10*	*Mamu*-KIR3DL10*005:01	3DL10-2DL501	GU014294	(Colantonio et al. 2011)
*Mamu*-*KIR3DL10*	*Mamu*-KIR3DL10*005:02	None	GU112295	(Blokhuis et al. 2010)
*Mamu*-*KIR3DL10*	*Mamu*-KIR3DL10*006	KIR3DL allele 6	FJ562113	(Bostik et al. 2009)
*Mamu*-*KIR3DL11*	*Mamu*-KIR3DL11*001	KIR3DL11	AF334626, GU112271	(Blokhuis et al. 2010; Hershberger et al. 2001)
*Mamu*-*KIR3DL11*	*Mamu*-KIR3DL11*002	KIR3DL-1	FN424250	(Kruse et al. 2010)
*Mamu*-*KIR3DL11*	*Mamu*-KIR3DL11*003	KIR3DL-6	FN424259	(Kruse et al. 2010)
*Mamu*-*KIR3DL11*	*Mamu*-KIR3DL11*004	KIR3DL-7	FN424261	(Kruse et al. 2010)
*Mamu*-*KIR3DL11*	*Mamu*-KIR3DL11*005	None	GU112276	(Blokhuis et al. 2010)
*Mamu*-*KIR3DL11*	*Mamu*-KIR3DL11*006	None	GU112296	(Blokhuis et al. 2010)
*Mamu*-*KIR3DL11*	*Mamu*-KIR3DL11*007	KIR3DL allele 9	FJ562116	(Bostik et al. 2009)
*Mamu*-*KIR3DL20*	*Mamu*-KIR3DL20*001	KIR3DL20*001-BNB	EU419047	(Moreland et al. 2011)
*Mamu*-*KIR3DL20*	*Mamu*-KIR3DL20*002	KIR3DL20	AY728184, GU112327	(Blokhuis et al. 2010; Sambrook et al. 2005)
*Mamu*-*KIR3DL20*	*Mamu*-KIR3DL20*003	KIR3DL20_variant_2	AY728186	(Sambrook et al. 2005)
*Mamu*-*KIR3DL20*	*Mamu*-KIR3DL20*004	KIR3DL20*003-BNB	EU419048	(Moreland et al. 2011)
*Mamu*-*KIR3DL20*	*Mamu*-KIR3DL20*005	KIR3DL20*004-BNB	EU419049	(Moreland et al. 2011)
*Mamu*-*KIR3DL20*	*Mamu*-KIR3DL20*006	None	GU112255	(Blokhuis et al. 2010)
*Mamu*-*KIR3DL20*	*Mamu*-KIR3DL20*007	None	GU112256	(Blokhuis et al. 2010)
*Mamu*-*KIR3DL20*	*Mamu*-KIR3DL20*008	None	GU112264	(Blokhuis et al. 2010)
*Mamu*-*KIR3DL20*	*Mamu*-KIR3DL20*009	None	GU112270	(Blokhuis et al. 2010)
*Mamu*-*KIR3DL20*	*Mamu*-KIR3DL20*010	None	GU112275	(Blokhuis et al. 2010)
*Mamu*-*KIR3DL20*	*Mamu*-KIR3DL20*011	None	GU112289	(Blokhuis et al. 2010)
*Mamu*-*KIR3DL20*	*Mamu*-KIR3DL20*012	None	GU112299	(Blokhuis et al. 2010)
*Mamu*-*KIR3DL20*	*Mamu*-KIR3DL20*013	None	GU112304, GU112317	(Blokhuis et al. 2010)
*Mamu*-*KIR3DL20*	*Mamu*-KIR3DL20*014	None	GU112308	(Blokhuis et al. 2010)
*Mamu*-*KIR3DL20*	*Mamu*-KIR3DL20*015	None	GU134802	(Blokhuis et al. 2010)
*Mamu*-*KIR3DS01*	*Mamu*-KIR3DS01*001:01	KIR3DH5	AF361087	(Grendell et al. 2001)
*Mamu*-*KIR3DS01*	*Mamu*-KIR3DS01*001:02	None	GU112307	(Blokhuis et al. 2010)
*Mamu*-*KIR3DS01*	*Mamu*-KIR3DS01*002	KIR3DH1	AY728190	(Sambrook et al. 2005)
*Mamu*-*KIR3DS01*	*Mamu*-KIR3DS01*003	KIR3DH-7	GU564161	(Chaichompoo et al. 2010)
*Mamu*-*KIR3DS02*	*Mamu*-KIR3DS02*001	KIR3DH2	AF334649	(Hershberger et al. 2001)
*Mamu*-*KIR3DS02*	*Mamu*-KIR3DS02*002	KIR3DH-like_5	AY505483	(Andersen et al. 2004)
*Mamu*-*KIR3DS02*	*Mamu*-KIR3DS02*003	KIR3DH-like_6	AY505484	(Andersen et al. 2004)
*Mamu*-*KIR3DS02*	*Mamu*-KIR3DS02*004:01	KIR3DH2*001-BNB, KIR3DH14	EU419026, EU702460	(Blokhuis et al. 2009a; Moreland et al. 2011)
*Mamu*-*KIR3DS02*	*Mamu*-KIR3DS02*004:02	KIR3DH13, 3DH42	EU702459, GU014296	(Blokhuis et al. 2009a) (Colantonio et al. 2011)
*Mamu*-*KIR3DS02*	*Mamu*-KIR3DS02*004:03	KIR3DH12	EU702458	(Blokhuis et al. 2009a)
*Mamu*-*KIR3DS02*	*Mamu*-KIR3DS02*005	KIR3DH2*002-BNB	EU419027	(Moreland et al. 2011)
*Mamu*-*KIR3DS02*	*Mamu*-KIR3DS02*006	KIR3DH16	EU702462	(Blokhuis et al. 2009a)
*Mamu*-*KIR3DS02*	*Mamu*-KIR3DS02*007	KIR3DH15	EU702461	(Blokhuis et al. 2009a)
*Mamu*-*KIR3DS02*	*Mamu*-KIR3DS02*008	KIR3DH10	EU702456, GU112278	(Blokhuis et al. 2009a; Blokhuis et al. 2010)
*Mamu*-*KIR3DS02*	*Mamu*-KIR3DS02*009	None	GU112261, GU112315	(Blokhuis et al. 2010)
*Mamu*-*KIR3DS02*	*Mamu*-KIR3DS02*010	None	GU112297	(Blokhuis et al. 2010)
*Mamu*-*KIR3DS02*	*Mamu*-KIR3DS02*011	None	GU112323	(Blokhuis et al. 2010)
*Mamu*-*KIR3DS02*	*Mamu*-KIR3DS02*012	3DH2*NEW1	JN613291	(Hellmann et al. 2011)
*Mamu*-*KIR3DS02*	*Mamu*-KIR3DS02*013	3DH2*NEW1	JN613299	(Hellmann et al. 2011)
*Mamu*-*KIR3DS03*	*Mamu*-KIR3DS03*001:01	KIR3DH3	AF334650, GU112312	(Hershberger et al. 2001) (Blokhuis et al. 2010)
*Mamu*-*KIR3DS03*	*Mamu*-KIR3DS03*001:02	None	GU112294	(Blokhuis et al. 2010)
*Mamu*-*KIR3DS03*	*Mamu*-KIR3DS03*002	KIR3DH9	EU702455, GU112269	(Blokhuis et al. 2009a; Blokhuis et al. 2010)
*Mamu*-*KIR3DS03*	*Mamu*-KIR3DS03*003	KIR3DH8	EU702454	(Blokhuis et al. 2009a)
*Mamu*-*KIR3DS04*	*Mamu*-KIR3DS04*001	KIR3DH4	AF334651	(Hershberger et al. 2001)
*Mamu*-*KIR3DS04*	*Mamu*-KIR3DS04*002	KIR3DH4*001-BNB	EU419028	(Moreland et al. 2011)
*Mamu*-*KIR3DS04*	*Mamu*-KIR3DS04*003	KIR3DH4*002-BNB, KIR3DH4	EU419029, JN613296	(Hellmann et al. 2011; Moreland et al. 2011)
*Mamu*-*KIR3DS04*	*Mamu*-KIR3DS04*004	KIR3DH6	EU702452	(Blokhuis et al. 2009a)
*Mamu*-*KIR3DS04*	*Mamu*-KIR3DS04*005	KIR3DH4	JN613300	(Hellmann et al. 2011)
*Mamu*-*KIR3DS04*	*Mamu*-KIR3DS04*006	KIR3DH-1	GU564157	(Chaichompoo et al. 2010)
*Mamu*-*KIR3DS05*	*Mamu*-KIR3DS05*001	KIR3DH1*001-BNB	EU419024, EU419025, EU702468, AY505487, GU112262	(Moreland et al. 2011)
*Mamu*-*KIR3DS05*	*Mamu*-KIR3DS05*002:01	KIR3DH1*002-BNB, KIR3DM1, KIR_Partial_Sequence_1	EU419025, EU702468, AY505487, GU112262	(Andersen et al. 2004; Blokhuis et al. 2009a; Blokhuis et al. 2010; Moreland et al. 2011)
*Mamu*-*KIR3DS05*	*Mamu*-KIR3DS05*002:02	KIR3DM6	EU702473	(Blokhuis et al. 2009a)
*Mamu*-*KIR3DS05*	*Mamu*-KIR3DS05*003	KIR3DM-1	FN424260	(Kruse et al. 2010)
*Mamu*-*KIR3DS06*	*Mamu*-KIR3DS06*001	KIR3DH-like_7	AY505485	(Andersen et al. 2004)
*Mamu*-*KIR3DS06*	*Mamu*-KIR3DS06*002:01	KIR3DH-like8	EU688985	(Moreland et al. 2011)
*Mamu*-*KIR3DS06*	*Mamu*-KIR3DS06*002:02	None	GU112298	(Blokhuis et al. 2010)
*Mamu*-*KIR3DS06*	*Mamu*-KIR3DS06*003	KIR3DH18	EU702464	(Blokhuis et al. 2009a)
*Mamu*-*KIR3DS06*	*Mamu*-KIR3DS06*004	KIR3DH-4	FN424257	(Kruse et al. 2010)
*Mamu*-*KIR3DS06*	*Mamu*-KIR3DS06*005	None	GU112260	(Blokhuis et al. 2010)
*Mamu*-*KIR3DS06*	*Mamu*-KIR3DS06*006	None	GU112314	(Blokhuis et al. 2010)
*Mamu*-*KIR3DSW07*	*Mamu*-KIR3DSW07*001	KIR3DH7	EU702453, GU112272	(Blokhuis et al. 2009a; Blokhuis et al. 2010)
*Mamu*-*KIR3DSW07*	*Mamu*-KIR3DSW07*002	KIR3DH-5	FN424258	(Kruse et al. 2010)
*Mamu*-*KIR3DSW08*	*Mamu*-KIR3DSW08*001	KIR3DH-like_1	AY505479	(Andersen et al. 2004)
*Mamu*-*KIR3DSW08*	*Mamu*-KIR3DSW08*002	KIR3DH-like_2	AY505480	(Andersen et al. 2004)
*Mamu*-*KIR3DSW08*	*Mamu*-KIR3DSW08*003	KIR3DH-like_3	AY505481	(Andersen et al. 2004)
*Mamu*-*KIR3DSW08*	*Mamu*-KIR3DSW08*004	KIR3DH-like_4	AY505482	(Andersen et al. 2004)
*Mamu*-*KIR3DSW08*	*Mamu*-KIR3DSW08*005	KIR3DH21	EU702467	(Blokhuis et al. 2009a)
*Mamu*-*KIR3DSW08*	*Mamu*-KIR3DSW08*006	KIR3DH-2	FN424254	(Kruse et al. 2010)
*Mamu*-*KIR3DSW08*	*Mamu*-KIR3DSW08*007	KIR3DH-3	FN424255	(Kruse et al. 2010)
*Mamu*-*KIR3DSW08*	*Mamu*-KIR3DSW08*008	None	GU112325	(Blokhuis et al. 2010)
*Mamu*-*KIR3DSW08*	*Mamu*-KIR3DSW08*009	None	GU112328	(Blokhuis et al. 2010)
*Mamu*-*KIR3DSW08*	*Mamu*-KIR3DSW08*010	KIR3DSW08	JN613297	(Hellmann et al. 2011)
*Mamu*-*KIR3DSW08*	*Mamu*-KIR3DSW08*011	KIR3DH-4	GU564158	(Chaichompoo et al. 2010)
*Mamu*-*KIR3DSW08*	*Mamu*-KIR3DSW08*012	KIR3DH-5	GU564159	(Chaichompoo et al. 2010)
*Mamu*-*KIR3DSW09*	*Mamu*-KIR3DSW09*001	KIR3DH5*001-BNB	EU419030	(Moreland et al. 2011)
*Mamu*-*KIR3DSW09*	*Mamu*-KIR3DSW09*002	KIR3DH5-like1	EU688986	(Moreland et al. 2011)
*Mamu*-*KIR3DSW09*	*Mamu*-KIR3DSW09*003	None	GU112301	(Blokhuis et al. 2010)
*Mamu*-*KIR3DSW09*	*Mamu*-KIR3DSW09*004	KIR3DH20	EU702466, GU112273	(Blokhuis et al. 2009a), (Blokhuis et al. 2010)
*Mamu*-*KIR3DSW09*	*Mamu*-KIR3DSW09*005	mmKIR3DH-1	FN424249	(Kruse et al. 2010)
*Mamu*-*KIR3DSW09*	*Mamu*-KIR3DSW09*006	KIR3DH-8	GU564162	(Chaichompoo et al. 2010)
*Mamu*-*KIR3DLX1*	*Mamu*-KIR3DLX1*001	KIR3DL0	DQ157756	(Sambrook et al. 2006)

**Table 5:** Allele designations and their previous names

Gene	Allele designation	Previous designations	Accession number	Reference
*Patr*-*KIR2DL4*	*Patr*-KIR2DL4*001	None	HM068617	(Abi-Rached et al. 2010)
*Patr*-*KIR2DL4*	*Patr*-KIR2DL4*002	None	AC155174, AF258804	(Khakoo et al. 2000)
*Patr*-*KIR2DL4*	*Patr*-KIR2DL4*003	None	BX842589	(Sambrook et al. 2005)
*Patr*-*KIR2DL5*	*Patr*-KIR2DL5*001	None	HM068617	(Abi-Rached et al. 2010)
*Patr*-*KIR2DL5*	*Patr*-KIR2DL5*002	None	AF274005	(Rajalingam et al. 2001)
*Patr*-*KIR2DL5*	*Patr*-KIR2DL5*003	None	AC155174	
*Patr*-*KIR2DL5*	*Patr*-KIR2DL5*004	None	BX842589	(Sambrook et al. 2005)
*Patr*-*KIR2DL5*	*Patr*-KIR2DL5*005	None	AF258805	(Khakoo et al. 2000)
*Patr*-*KIR2DL6*	*Patr*-KIR2DL6*001	None	BX842589, AM292662	(Sambrook et al. 2005)
*Patr*-*KIR2DL6*	*Patr*-KIR2DL6*002	None	AF258806	
*Patr*-*KIR2DL6*	*Patr*-KIR2DL6*003	None	AM292661	
*Patr*-*KIR2DL7*	*Patr*-KIR2DL7*001	None	HM068617	(Abi-Rached et al. 2010)
*Patr*-*KIR2DL8*	*Patr*-KIR2DL8*001	None	HM068617	(Abi-Rached et al. 2010)
*Patr*-*KIR2DL8*	*Patr*-KIR2DL8*002	None	AC155174, AM279149	Biassoni, unpublished
*Patr*-*KIR2DL8*	*Patr*-KIR2DL8*003	None	BX842589	(Sambrook et al. 2005)
*Patr*-*KIR2DL9*	*Patr*-KIR2DL9*001	None	AC155174	
*Patr*-*KIR2DL9*	*Patr*-KIR2DL9*002	None	AM292657	Biassoni, unpublished
*Patr*-*KIR2DL9*	*Patr*-KIR2DL9*003	None	AM400233	Biassoni, unpublished
*Patr*-*KIR2DS4*	*Patr*-KIR2DS4*001	None	HM068617	
*Patr*-*KIR2DS4*	*Patr*-KIR2DS4*002	None	AF258807	
*Patr*-*KIR3DL1*	*Patr*-KIR3DL1*001:01	None	AC155174	
*Patr*-*KIR3DL1*	*Patr*-KIR3DL1*001:02	None	AF266729	(Rajalingam et al. 2001)
*Patr*-*KIR3DL1*	*Patr*-KIR3DL1*002	None	BX842589, AF258798	(Sambrook et al. 2005)
*Patr*-*KIR3DL1*	*Patr*-KIR3DL1*003	None	AF266730	(Rajalingam et al. 2001)
*Patr*-*KIR3DL1*	*Patr*-KIR3DL1*004	None	AF258799	
*Patr*-*KIR3DL1*	*Patr*-KIR3DL1*005	None	HM068617	
*Patr*-*KIR3DL3*	*Patr*-KIR3DL3*001	None	HM068617	
*Patr*-*KIR3DL3*	*Patr*-KIR3DL3*002	None	BX842589	
*Patr*-*KIR3DL3*	*Patr*-KIR3DL3*003	None	AC155174	
*Patr*-*KIR3DL3*	*Patr*-KIR3DL3*004	None	AY327500	
*Patr*-*KIR3DL4*	*Patr*-KIR3DL4*001:01	None	AM400232	Biassoni, unpublished
*Patr*-*KIR3DL4*	*Patr*-KIR3DL4*001:02	None	AF258800	(Khakoo et al. 2000)
*Patr*-*KIR3DL4*	*Patr*-KIR3DL4*002	None	HM068617	(Abi-Rached et al. 2010)
*Patr*-*KIR3DL5*	*Patr*-KIR3DL5*001	None	AM400235	Biassoni, unpublished
*Patr*-*KIR3DL5*	*Patr*-KIR3DL5*003:01	None	AF258801	(Khakoo et al. 2000)
*Patr*-*KIR3DL5*	*Patr*-KIR3DL5*004	None	AC155174, AM292659	Biassoni, unpublished
*Patr*-*KIR3DS2*	*Patr*-KIR3DS2*001	None	AC155174	
*Patr*-*KIR3DS2*	*Patr*-KIR3DS2*002	None	AF258803	
*Patr*-*KIR3DS6*	*Patr*-KIR3DS6*001	None	AM396937	Biassoni, unpublished

**Table 6:** Allele designations and their previous names

Gene	Allele designation	Previous designations	Accession number	Reference
*Poab*-*KIR2DL10*	*Poab*-KIR2DL10*001	2DLA	AF470358	(Guethlein et al. 2002)
*Poab*-*KIR2DL11*	*Poab*-KIR2DL11*001	2DLB	EF014479	(Guethlein et al. 2007b)
*Poab*-*KIR2DL12*	*Poab*-KIR2DL12*001	2DLC	AC200148	
*Poab*-*KIR2DL5*	*Poab*-KIR2DL5*001	2DL5	AC200148	
*Poab*-*KIR2DS10*	*Poab*-KIR2DS10*001	None	AF470364	(Guethlein et al. 2002)
*Poab*-*KIR2DS13*	*Poab*-KIR2DS13*001	2DSC1/2DSB	AF470362	(Guethlein et al. 2002)
*Poab*-*KIR2DS14*	*Poab*-KIR2DS14*001	2DSB/2DSD2	AF470361	(Guethlein et al. 2002)
*Poab*-*KIR2DS14*	*Poab*-KIR2DS14*002	2DSA/2DSD1	AF470360	(Guethlein et al. 2002)
*Poab*-*KIR3DL1*	*Poab*-KIR3DL1*001:01	3DLH	AF470373	(Guethlein et al. 2002)
*Poab*-*KIR3DL1*	*Poab*-KIR3DL1*001:02	None	AC200148	
*Poab*-*KIR3DL1*	*Poab*-KIR3DL1*002	3DLC	AF470367	(Guethlein et al. 2002)
*Poab*-*KIR3DL1*	*Poab*-KIR3DL1*003	None	AF470372	(Guethlein et al. 2002)
*Poab*-*KIR3DL1*	*Poab*-KIR3DL1*004:01	3DLD2	AF470369	(Guethlein et al. 2002)
*Poab*-*KIR3DL1*	*Poab*-KIR3DL1*004:02	3DLD1	EF014479	(Guethlein et al. 2007b)
*Poab*-*KIR3DL1*	*Poab*-KIR3DL1*005	3DLA	AF470365	(Guethlein et al. 2002)
*Poab*-*KIR3DL1*	*Poab*-KIR3DL1*006	3DLI	AF470374	(Guethlein et al. 2002)
*Poab*-*KIR3DL1*	*Poab*-KIR3DL1*007	3DLB	AF470366	(Guethlein et al. 2002)
*Poab*-*KIR3DL3*	*Poab*-KIR3DL3*001	3DL3	AC200148	
*Poab*-*KIR3DS1*	*Poab*-KIR3DS1*001	3DS1	AF470375	(Guethlein et al. 2002)
*Poab*-*KIRDP*	*Poab*-KIRDP*001	DP	AC200148	
*Popy*-*KIR2DS10*	*Popy*-KIR2DS10*001	2DSD/2DSA	AF470364	(Guethlein et al. 2002)
*Popy*-*KIR2DS13*	*Popy*-KIR2DS13*001	2DSC2/2DSB	AF470363	(Guethlein et al. 2002)
*Popy*-*KIR3DL1*	*Popy*-KIR3DL1*001	3DLF	AF470372	(Guethlein et al. 2002)
*Popy*-*KIR3DL1*	*Popy*-KIR3DL1*002:01	3DLE2	AF470371	(Guethlein et al. 2002)
*Popy*-*KIR3DL1*	*Popy*-KIR3DL1*002:02	3DLE1	AF470370	(Guethlein et al. 2002)

**Table 7:** Allele designations and their previous names

Gene	Allele designation	Previous designations	Accession number	Breed	Reference
*Bota*-*KIR2DL1*	*Bota*-KIR2DL1*001	KIR2DL1	AY075102,AF490399	UnknownHolstein	(McQueen et al. 2002; Storset et al. 2003; Zimin et al. 2009)
*Bota*-*KIR2DL1*	*Bota*-KIR2DL1*002	None	JX848327	Holstein-Freisian	(Sanderson et al. 2014)
*Bota*-*KIR2DS1*	*Bota*-KIR2DS1*001N	KIR2DS1	JX848328	Holstein-Freisian	(Sanderson et al. 2014)
*Bota*-*KIR2DS2*	*Bota*-KIR2DS2*001N	None	JX848329	Holstein-Freisian	(Sanderson et al. 2014)
*Bota*-*KIR2DS3*	*Bota*-KIR2DS3*001N	None	JX848330	Holstein-Freisian	(Sanderson et al. 2014)
*Bota*-*KIR2DXS1*	*Bota*-KIR2DXS1*001	None	AF490400	Holstein	(Storset et al. 2003)
*Bota*-*KIR2DXP1*	*Bota*-KIR2DXP1*001	None	JX848331	Holstein-Freisian	(Sanderson et al. 2014)
*Bota*-*KIR2DXP2*	*Bota*-KIR2DXP2*001	None	JX848332	Holstein-Freisian	(Sanderson et al. 2014)
*Bota*-*KIR3DXL1*	*Bota*-KIR3DXL1*001	KIR3DL1	AF490402	Holstein	(Storset et al. 2003; Zimin et al. 2009)
*Bota*-*KIR3DXL1*	*Bota*-KIR3DXL1*002	None	JX848333	Holstein-Freisian	(Sanderson et al. 2014)
*Bota*-*KIR3DXL2*	*Bota*-KIR3DXL2*001	None	JX848334	Holstein-Freisian	(Sanderson et al. 2014)
*Bota*-*KIR3DXL3*	*Bota*-KIR3DXL3*001	None	JX848335	Holstein-Freisian	(Sanderson et al. 2014)
*Bota*-*KIR3DXL4*	*Bota*-KIR3DXL4*001	KIR3DL2–001	EF197118	Holstein-Freisian	(Dobromylskyj and Ellis 2007; Zimin et al. 2009)
*Bota*-*KIR3DXL4*	*Bota*-KIR3DXL4*002	None	JX848336	Holstein-Freisian	(Sanderson et al. 2014)
*Bota*-*KIR3DXL5*	*Bota*-KIR3DXL5*001	None	JX848337	Holstein-Freisian	(Sanderson et al. 2014)
*Bota*-*KIR3DXL6*	*Bota*-KIR3DXL6*001N	KIR3DL1P	AY075103JX848338	UnknownHolstein-Freisian	(McQueen et al. 2002) (Sanderson et al. 2014)
*Bota*-*KIR3DXL6*	*Bota*-KIR3DXL6*002	KIR3DL3	EF197119	Holstein-Freisian	(Dobromylskyj and Ellis 2007; Zimin et al. 2009)
*Bota*-*KIR3DXL7*	*Bota*-KIR3DXL7*001	None	JX848339	Holstein-Freisian	(Sanderson et al. 2014)
*Bota*-*KIR3DXS1*	*Bota*-KIR3DXS1*001	KIR3DS1	AF490401	Holstein	(Storset et al. 2003; Zimin et al. 2009)
*Bota*-*KIR3DXS1*	*Bota*-KIR3DXS1*002	KIR3DS1–002	EF197120	Holstein-Freisian	(Dobromylskyj and Ellis 2007)
*Bota*-*KIR3DXS1*	*Bota*-KIR3DXS1*003	None	JX848340	Holstein-Freisian	(Sanderson et al. 2014)
*Bota*-*KIR3DXS2*	*Bota*-KIR3DXS2*001N	None	JX848341	Holstein-Freisian	(Sanderson et al. 2014)
*Bota*-*KIR3DXS3*	*Bota*-KIR3DXS3*001N	None	JX848342	Holstein-Freisian	(Sanderson et al. 2014)

Each *KIR* allele name includes a unique number corresponding to up to three sets of digits separated by colons. All alleles are given a three-digit name, which corresponds to the first set of digits; longer names are assigned only when necessary.

The digits placed before the first colon describe the alleles that differ at non-synonymous substitutions (also called coding substitutions). Alleles that differ only by synonymous nucleotide substitutions (also called silent or non-coding substitutions) but are within the coding sequence are distinguished by their second sets of digits. Alleles that only differ by sequence polymorphisms in the introns, or in the 5′ or 3′ untranslated regions that flank the exons and introns, are distinguished by their third sets of digits.

In addition to the unique allele number, optional suffixes can be added to an allele name to indicate the expression status of the gene and/or its encoded protein. Alleles known not to be expressed—so called “Null” alleles—have been given the suffix “N.” Alleles that have been shown to be alternatively expressed may have the suffix “L,” “S,” “C,” “A,” or “Q.”

The suffix “L” is used to indicate an allele that has been shown to have “Low” cell surface expression when compared to normal levels. The “S” suffix is used to denote an allele specifying a protein which is expressed as a soluble, “Secreted” molecule and is not present on the cell surface. The “C” suffix is assigned to alleles producing proteins that are present in the “Cytoplasm” and not on the cell surface. An “A” suffix indicates an “Aberrant” expression, where there is doubt as to whether a protein is actually expressed. A “Q” suffix is used when the expression of an allele is “Questionable,” given that the mutation seen in the allele has been shown to affect normal expression levels in other alleles and other KIR genes.

As of May 2018, no alleles have been named with the “C,” “A,” “Q,” or “S” suffixes.

**Fig. 1:**
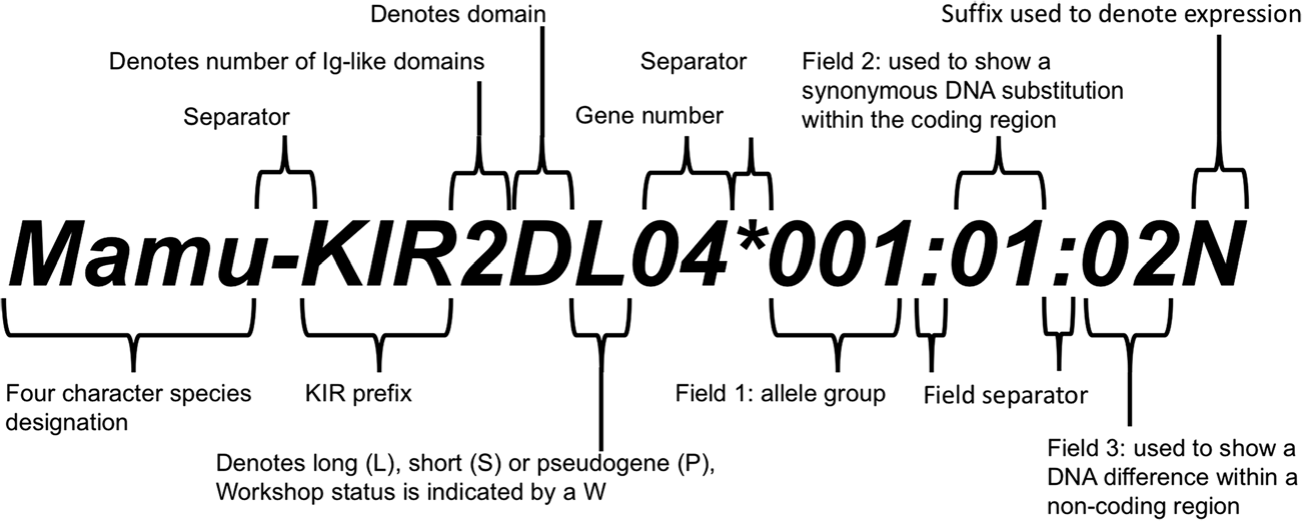
Non-human KIR nomenclature. Details the syntax and structure of a non-human KIR allele designation

A schematic representation of the syntax for the non-human KIR allele designation is shown in Fig. 1.

### Species-specific guidelines

#### Naming rhesus macaque *KIR* genes

The *Mamu*-*KIR* sequences fall into a number of distinct lineages based on phylogenetic analysis. Most sequences correspond to lineage II *KIR* and are further divided into those encoding KIR that have long cytoplamic tails or short cytoplasmic tails. The genes have been numbered sequentially and where possible the gene name has the same the same number as the first reported allele for that gene. For example, the *Mamu*-*KIR3DL1* gene (Hershberger et al. 2001) was renamed *Mamu*-KIR3DL01*001.

The nomenclature uses a two-digit numbering of individual genes for the macaque sequences as seen with the naming of *Mamu*-KIR3DL01*001. This renaming aims to avoid confusion with previous sequence names. Subsequent analysis has shown that some of the proposed sequences of different genes are actually allelic variants of the same gene. Rather than skipping numbers to avoid confusion, it was thought better to introduce the two-digit numbering system.

Recombinant alleles are named according to the locus, which provide the majority of the sequence. For example, the sequence originally named *Mamu*-*KIR3DL5* (Hershberger et al. 2001) is a recombinant of *Mamu*-*KIR3DL01* and *Mamu*-*KIR3DL07*. As such, it has been renamed as an allele of *Mamu*-*KIR3DL01*, *Mamu*-KIR3DL01*005. This principal has also been applied to recombinant alleles in other species.

Along with the lineage II KIR genes, rhesus macaques have *KIR* genes for lineage I, III, and V *KIR*. The lineage I *KIR* gene in rhesus macaques is orthologous to other primate lineage 1 KIR, referred to as *2DL4* and has been named *Mamu*-*KIR2DL04*. A single lineage III KIR is also present on some *Mamu*-*KIR* haplotypes and in all cases appears to be expressed as a one Ig domain KIR. It has been named *Mamu*-*KIR1D*. Finally, there is a lineage V KIR gene that is expressed as either a two Ig or three Ig domain KIR. The published genomic sequence shows the gene to contain three Ig domain encoding exons; however, due to splicing out of exon 4, also two Ig domain KIR variants are expressed. The majority of the rhesus macaque gene sequence appears orthologous to hominoid *KIR3DL3* sequences, the exception being exon 3 [encoding the D0 domain] which appears more like the hominoid *KIR2DL5* sequences. This sequence relationship coupled with the presence of splice variants that lacked exon 4 led to the naming of some of these sequences as *Mamu*-*KIR2DL5*. The presence of the intact gene as evidenced by the published genomic sequence, as well as the existence of full-length [three Ig domain containing] sequences has led us to propose naming this gene as *Mamu*-*KIR3DL20*. This distinguishes this gene from the remaining Mamu-KIR3DL as well as retaining the name of one of the first mRNA sequences that included all three Ig domain encoding exons, see Table 1 for further details. A full list of *Mamu*-*KIR* sequences is described in Table 4.

The identification of sequences in other Macaque species will follow the same rules, and use the species prefix (Mafa-KIR, Mane-KIR), and that genes would be named to match the closest rhesus gene.

#### Naming chimpanzee *KIR* genes

Three studies (Abi-Rached et al. 2010; Khakoo et al. 2000; Sambrook et al. 2005) have described complete sequences of three chimpanzee haplotypes. In addition, the analysis of chimpanzee *KIR* genotypes has inferred the organization of genes infers the existence of another 17 chimpanzee *KIR* haplotypes. These analyses have defined 13 different *Patr*-*KIR* genes.

In all chimpanzee *KIR* haplotypes, the framework gene at the telomeric end is a lineage II *KIR* gene. Formerly, two variants, now known to occupy this position, were named *Pt*-*KIR3DL1*/*2* and *Pt*-*KIR3DL3*. The name *Pt*-*KIR3DL1*/*2* was given to reflect its close relationship to both human *KIR3DL1* and *KIR3DL2*. Although segregation analysis showed that *Pt*-*KIR3DL3* and *KIR3DL1*/*2* were never present on the same haplotype, *Pt*-*KIR3DL3* was given a different name because it has a distinctive sequence. We are renaming the *Pt*-*KIRDl1*/*2* and *Pt*-*KIR3DL3* as allelic variants of *Patr*-*KIR3DL1*, the new name for the framework gene at the telomeric end of the chimpanzee *KIR* locus. This will allow the *Patr*-*KIR3DL3* name to be given to the gene previously known as *Patr*-*KIRC1*, and which is orthologous to human *KIR3DL3*, the framework gene at the centromeric end of the *KIR* locus. See Table 2 for further details. A full list of *Patr*-*KIR* sequences is described in Table 5.

#### Naming orangutan *KIR* genes

In the initial description of orangutan *KIR* cDNA (Guethlein et al. 2002), the sequences were given letter designations because their relationships, either alleles or genes, were uncertain. Subsequent studies (Guethlein et al. 2007a; Guethlein et al. 2017; Locke et al. 2011; Mager et al. 2001) have provided complete sequences of three orangutan *KIR* haplotypes, as well as genotyping data that has allowed the structures of two additional *KIR* haplotypes to be inferred. These genomic data, in combination with the cDNA sequences, defined 11 *KIR* genes and 1 *KIR* pseudogene in the orangutan. At first, all orangutan *KIR* were named as “Popy” (Guethlein et al. 2007b). The orangutan *KIR* is now divided into two series corresponding to the two species of orangutan: Popy for *Pongo pygmaeus* and Poab for *Pongo abelii* depending on species of origin. Some *KIR* alleles are present in both orangutan species. These alleles shared have been given a different name in each species (Guethlein et al. 2017; Guethlein et al. 2015), see Table 3: for further details. A full list of *Popy*-*KIR* and *Poab*-*KIR* sequences is given in Table 6.

#### Naming cattle *KIR* genes

Assembly of the first cattle *KIR* haplotype allowed previously known cDNA sequences to be assigned to particular genes and allelic relationships to be defined (Dobromylskyj and Ellis 2007; Guethlein et al. 2007a; Hammond et al. 2016; Mager et al. 2001; Sanderson et al. 2014). This presents the opportunity to adopt an accurate and logical nomenclature system. Cattle *KIR* cDNA sequences were previously named using the established convention of Ig domain number and tail length. However, these alleles were annotated prior to the discovery of a second deeply divergent *KIR* lineage, the *KIR3DX* lineage (Guethlein et al. 2007a). The majority of the expanded cattle *KIR* belong to this second lineage. In developing a nomenclature system for the cattle *KIR*, we have incorporate their lineage ancestry within the name. Cattle *KIR* have been prefixed with a four-letter species designation “Bota” (*Bos taurus*) in line with non-human primates. Where possible previously named *Bota*-*KIR* has retained the same name with only the addition of an “X” after the domain number if from the *KIR3DX* lineage. There are three exceptions; *Bota*-*KIR3DL1P* and *Bota*-*KIR3DL3*, which are allelic, and *Bota*-*KIR3DL2*. These previously described cDNA sequences are all members of the *KIR3DX* lineage. Based on their position in the cattle haplotype and their relationships to other genes, *Bota*-*KIR3DL1P* was renamed *Bota*-KIR3DXL6*001N, *Bota*-*KIR3DL3* was renamed *Bota*-KIR3DXL6*002, and *Bota*-*KIR3DL2* was renamed *Bota*-*KIR3DXL4*. We have identified 16 cattle *KIR* genes. The proposed nomenclature for cattle *KIR* is given in Table 7.

#### Future guidelines

The sequences described in this report will be included in the Immuno Polymorphism Database (IPD) (Robinson et al. 2013). They will be maintained as a component of the IPD and be accessible at https://www.ebi.ac.uk/ipd/nhkir/. New sequences for any of the above species can be submitted using the current submission tool. As with the other databases, there are requirements that should be met before formal names can be given and the submitted KIR are included in the database. First, submission of full-length sequences is encouraged and for some species like rhesus macaque is already mandatory. Second, novel sequences must be confirmed, either through their replication in multiple individuals or at a minimum by coming from multiple independent PCR/cloning experiments. Full guidelines for submission of non-human KIR sequences to IPD can be found at https://www.ebi.ac.uk/ipd/nhkir/submission/help.

As KIR sequence data from other species reaches the level of the species included in this report, those species can be included in the database. The inclusion of a species will be at the discretion of the Nomenclature Committee and IPD and will be based on the number of sequences available as well as evidence of identified genes and haplotype structure.
